# Tissue Engineering of Corneal Endothelium

**DOI:** 10.3390/jfb3040726

**Published:** 2012-10-17

**Authors:** Tatsuya Mimura, Seiichi Yokoo, Satoru Yamagami

**Affiliations:** 1 Department of Ophthalmology, Tokyo Women’s Medical University Medical Center East, 2-1-10 Nishiogu, Arakawa-ku, Tokyo 116-8567, Japan; 2 Department of Ophthalmology, University of Tokyo Graduate School of Medicine, 7-3-1 Hongo, Bunkyo-ku, Tokyo 113-8655, Japan; Email: syokoo-tky@umin.ac.jp (S.Y.); syamagami-tky@umin.ac.jp (S.Y.)

**Keywords:** review, corneal endothelium, descemet stripping automated endothelial keratoplasty, tissue engineering, transplantation

## Abstract

Human corneal endothelial cells (HCECs) do not replicate after wounding. Therefore, corneal endothelial deficiency can result in irreversible corneal edema. Descemet stripping automated endothelial keratoplasty (DSAEK) allows selective replacement of the diseased corneal endothelium. However, DSAEK requires a donor cornea and the worldwide shortage of corneas limits its application. This review presents current knowledge on the tissue engineering of corneal endothelium using cultured HCECs. We also provide our recent work on tissue engineering for DSAEK grafts using cultured HCECs. We reconstructed DSAEK grafts by seeding cultured DiI-labelled HCECs on collagen sheets. Then HCEC sheets were transplanted onto the posterior stroma after descemetorhexis in the DSAEK group. Severe stromal edema was detected in the control group, but not in the DSAEK group throughout the observation period. Fluorescein microscopy one month after surgery showed numerous DiI-labelled cells on the posterior corneal surface in the DSAEK group. Frozen sections showed a monolayer of DiI-labelled cells on Descemet’s membrane. These findings indicate that cultured adult HCECs, transplanted with DSAEK surgery, maintain corneal transparency after transplantation and suggest the feasibility of performing DSAEK with HCECs to treat endothelial dysfunction.

## 1. Introduction

**Figure 1:**
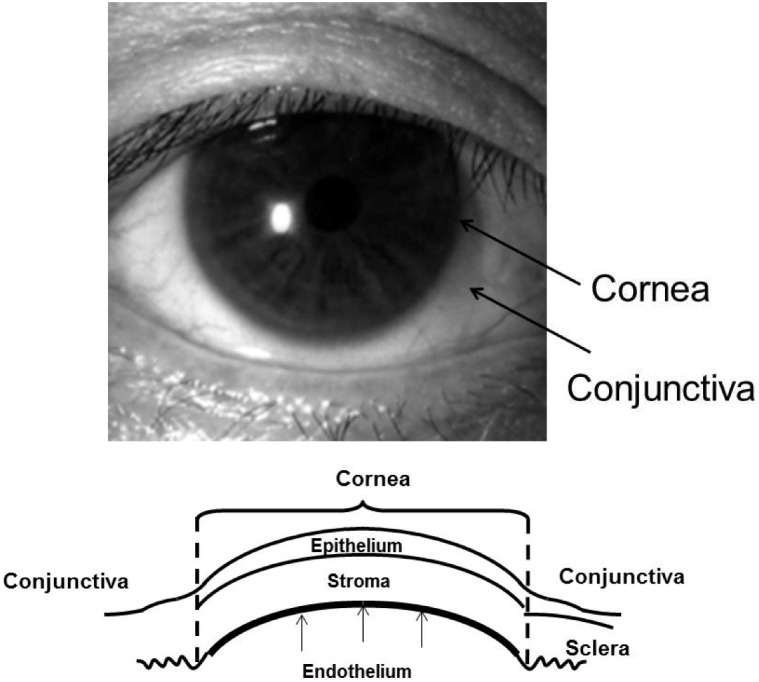
Anterior view of a human cornea and a diagram of the corneal epithelium, stroma and endothelium.

The cornea is composed of a multilayered epithelium, Bowman’s membrane, stroma, Descemet’s membrane, and endothelium (Figure 1). Corneal endothelial cells (CECs) are firmly attached to the underlying Descemet’s membrane. Corneal endothelial cells are believed to be of neural crest origin [1,2] and form a monolayer of hexagonal cells. Transparency of the cornea is maintained by regulation of stromal hydration through the barrier and pump functions of the corneal endothelium. Human CECs (HCECs) normally have a limited proliferative capacity *in vivo* [3,4,5,6,7] because they are maintained in a Gl-phase arrested state [8,9]. Therefore, HCECs gradually decrease with age throughout life [10,11,12].

When primarily damaged, it is reasonable to replace only the corneal endothelium. Due to technical difficulties, treatment for corneal endothelial decompensation, such as pseudophakic or aphakic bullous keratopathy and Fuchs dystrophy, has been limited to penetrating keratoplasty (PKP). However, descemet stripping with automated endothelial keratoplasty (DSAEK) has recently become a standard procedure for corneal transplantation in patients with endothelial dysfunction [13,14,15,16]. This procedure improves postoperative visual function and reduces the risks associated with PKP, such as severe astigmatism and expulsive hemorrhage. However, DSAEK still requires a fresh donor cornea and endothelium, which are in limited supply.

To overcome the problems of a donor cornea shortage, the application of CEC sheet transplantation using cultured HCECs has been attempted in experimental studies as a substitute for full-thickness corneal transplantation. Cultured HCECs derived from adult human donor corneas have been transplanted onto the denuded Descemet’s membrane [17,18,19,20,21,22,23,24,25,26,27], collagen matrix [28], amniotic membrane [29], human corneal stromal discs [30,31], gelatin hydrogel discs [32,33], and chitosan-based membranes [34] *ex vivo*.

In this review, we will discuss the feasibility of DSAEK using cultured HCECs, as well as our recent work on the transplantation of cultured HCECs [28,30]. In the study described here, all procedures were performed in accordance with the ARVO Statement for the Use of Animals in Ophthalmic and Vision Research. Human donor corneas were handled according to the tenets of the Declaration of Helsinki of 1975 and its 1983 revision.

## 2. Culture Technique of Human Corneal Endothelial Cells

### 2.1. Extracellular Matrix Substrates for Human Corneal Endothelial Cell Culture

Several groups have established HCEC culture techniques [21,24,29,35,36]. It is reported that the attachment and growth of cultured HCECs can be promoted by artificial matrices, such as chondroitin sulfate and laminin [37], laminin-5 [38], extracellular matrix secreted by bovine corneal endothelial cells [36,39], and fibronectin plus type I collagen coating mix [40].

Recently, our laboratory investigated the expression of laminin-5 (LM5) and its receptors in HCECs and the influence of recombinant human LM5 on the adhesion, proliferation, and migration of cultured HCECs [38]. Human CECs expressed the LM5 receptor α3β1 integrin. Recombinant LM5 promoted adhesion, migration, and moderate proliferation of cultured HCECs. These results suggest that the functional system involving LM5 and the α3β1 receptor for LM5 may be a critical factor in promoting HCEC culture and may contribute to the practical use of tissue-engineered HCECs.

### 2.2. Growth Factors and Cytokines for Human Corneal Endothelial Cell Culture

In addition, various growth factors have been reported to influence the proliferation of cells cultured from human corneal endothelium, including fibroblast growth factor [21,24,35,37,41,42], epidermal growth factor [24,35,42,43], nerve growth factor [24], and endothelial cell growth supplement [35,39].

Recently, our laboratory developed a simple culture method for HCECs using L-ascorbic acid 2-phosphate (Asc-2P) [44]. In the study, the influence of Asc-2P on the growth of cultured HCECs was examined. Culturing with Asc-2P and basic fibroblast growth factor (bFGF) in atelocollagen promoted the proliferation of HCECs in both primary cultures and subcultures. During multiple passages, cultures without Asc-2P showed a decrease in growth and irregular cell morphology, whereas cultures with Asc-2P sustained cell growth and maintained the characteristic polygonal morphology. The levels of 8-hydroxy-2-deoxyguanosine in mitochondrial DNA showed a significant decrease when HCECs were subcultured with Asc-2P. These results suggest that the combination of Asc-2P and bFGF in atelocollagen allows successful culturing of HCECs. We speculated that Asc-2P extends the lifespan of cultured HCECs, partly due to its protection against oxidative DNA damage. Although many details of the mechanisms by which Asc-2P promotes cell growth remain unknown, the cell growth seems to be mediated through the scavenging of reactive oxygen species (ROS) and regulation of the synthesis of proteins related to cell growth. Joyce and associates demonstrated that treatment of cultured HCECs from young donors with increasing concentrations of hydrogen peroxide causes a dose-dependent increase in nuclear 8-hydroxy-2’-deoxyguanosine staining and a decrease in proliferative capacity similar to that observed in untreated HCECs from older donors [45]. They concluded that age-dependent and topographical decreases in proliferative capacity observed in HCECs resulted, at least in part, from nuclear oxidative DNA damage [45]. Conversely, hypoxia stimulates the growth of various cells [46,47], along with a decrease of intracellular ROS [48] and decreased expression of a negative cell cycle regulator, p21 Cip1 [49]. Taken together with our data indicating that Asc-2P potently diminished intracellular ROS generation, Asc-2P may promote HCEC growth by reducing intracellular oxidative stress.

Recently, Mergler and associates examined gene expression and function of transient receptor potential (TRP) channels of the vanilloid (V) isoform subfamily (*i.e.*, TRPV1–3) in cultured HCECs to prove the role of temperature sensitive ion channels of cultured HCECs [50,51]. They demonstrated the expression of TRPV isotypes 1, 2 and 3 were detected by RT-PCR and protein expression of TRPV1 *in situ* was confirmed by immunostaining of corneoscleral remnants after keratoplasty. TRPV1-3 functional activity was evident based on capsaicin-induced Ca^2+^ transients and induction of these responses through rises in ambient temperature from 25 °C to over 40 °C. The currents underlying Ca^2+^ transients were characterized with a novel high throughput patch-clamp system. The TRPV1 selective agonist, capsaicin (10–20 µM) increased non-selective cation whole-cell currents resulting in calcium increases that were fully blocked by either the TRPV1 antagonist capsazepine or removal of extracellular calcium. Similarly, heating from room temperature to over 40 °C increased the same currents resulting in calcium increases that were significantly reduced by the TRP channel blockers lanthanum chloride^3+^ (100 µM) and ruthenium-red (10 µM), respectively. Moreover, application of the TRPV channel opener 2-aminoethoxydiphenyl borate (400 µM) led to a reversible increase in intracellular Ca^2+^ indicating putative TRPV1–3 channel activity. From these results, they concluded that TRPV activity modulation by temperature underlies essential homeostatic mechanisms contributing to the support of corneal endothelial function under different ambient conditions [50,51].

### 2.3. Overview of Human Corneal Endothelial Cell Culture

Currently, various culture media are used for cultivating HCECs, including Dulbecco’s modified Eagle’s medium (DMEM), Opti-MEM-I, DMEM/F12, and Ham’s F12/M199, all of which have given satisfactory results. Peh and associates evaluated the effect of these four culture media in the isolation and propagation of HCECs [52]. They cultured HCECs in these four medium and found that HCECs isolated in all four media showed rapid attachment when cultured on FNC-coated dishes. However, HCECs cultured in DMEM and DMEM/F12 could not be propagated beyond the first and second passage, respectively. The HCECs cultured in Opti-MEM-I and Ham’s F12/M199 were significantly more proliferative. However, the morphological characteristics of cultured HCECs were not maintained in either Opti-MEM-I or Ham’s F12/M199 beyond the third passage. They concluded that the HCECs cultured in Opti-MEM-I and Ham’s F12/M199 were significantly more proliferative and expressed markers characteristic of human corneal endothelium: Na+K+/ATPase and ZO-1 [52].

Furthermore, Engelmann’s laboratory also recently evaluated the influence of five different organ culture media on corneal endothelial cell survival [53]. They cultured HCECs in five different media: HCEC growth medium (F99), standard MEM containing 2% fetal calf serum (FCS), MEM containing 5% FCS, and humanized, endothelial serum-free medium (SFM) (with and without antibiotics). They found that the number of apoptotic cells in untreated control cultures was significantly higher in MEM compared with F99 and SFM. They concluded that HCECs cultured in MEM media seem susceptible to cell death in the absence of exogenous noxious stimuli, while HCEC cultured in SFM seem to be protected from cell death even when the apoptosis-inducer staurosporine is added [53]. They also speculated that the nutritive conditions in MEM therefore seem to be insufficient to sustain adequate compensatory mechanisms against oxidative cell stress. 

### 2.4. Human Corneal Endothelial Cell Culture in Our Laboratory

**Figure 2:**
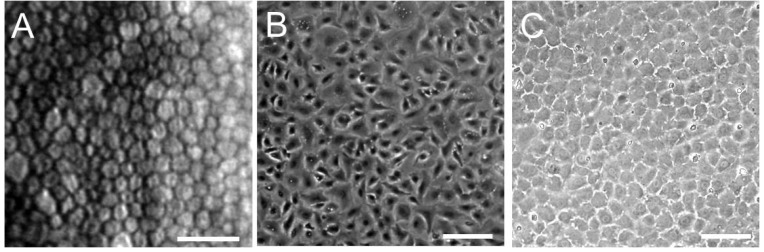
Specular microscopy of a 62-year-old male patient (**A**) and semi-confluent;and (**B**) full-confluent; (**C**) P6 cultured human corneal endothelial cells (HCECs) derived from a 65-year-old donor. Confluent cells show the characteristic hexagonal shape of corneal endothelial cells. Scale bar = 100 µm.

Here, we describe a general culture method for HCEC that was performed in our laboratory. This method is based on both the published protocols of Joyce and our laboratory, with some modifications [24,36,40,54,55]. In our laboratory, all donor corneas were obtained from the Rocky Mountain Lion’s Eye Bank. Briefly, Descemet’s membrane with intact endothelium was carefully dissected. After centrifugation, the strips were incubated in 0.02% ethylenediamine tetraacetic acid disodium salt solution at 37 °C for 1 h to loosen intercellular junctions. Isolated cells were plated in 6-well tissue culture plates precoated with undiluted fibronectin plus type I collagen coating mix. The cultures were then maintained in Opti-MEM-I or low-glucose DMEM containing 8% fetal bovine serum and 2 ng/mL bFGF. Joyce’s laboratory used a culture medium (OptiMEM-I) supplemented with 8% fetal bovine serum, 5 ng/mL epidermal growth factor (EGF), 20 ng/mL nerve growth factor (NGF), 100 μg/mL bovine pituitary extract, 20 μg/mL ascorbic acid, 200 mg/mL calcium chloride, and 0.08% chondroitin sulfate for HCEC culture [55]. The plates were then incubated at 37 °C in a humidified atmosphere with 5% carbon dioxide. Once cells reached confluence, they were cultured in medium without supplements, such as FGF, EGF, NGF, or pituitary extract, for several days in order to stabilize the monolayer and optimally reflect its *in vivo* morphology [55]. After primary cultures reached confluence, cells were subcultured at a 1:4 ratio. With this method, primary cultured HCECs usually reached confluence in seven to ten days. Moreover, even the cultured HCECs at Passage 6 or 7 obtained from adult donor corneas by this procedure retained the HCEC-like morphology, exhibiting a regular hexagonal morphology *in vivo* (Figure 2).

Recently, we succeeded in culturing and maintaining HCECs in a serum-free condition [38,44]. The use of serum-free medium avoids possible contamination with viruses, zoonosis from animals, and infection from bovine diseases by prions. Considering the clinical application of HCEC sheets, we must solve various problems, such as how to maintain product quality of culture medium during culturing HCECs and how to remove all animal-derived components from HCEC culture medium.

## 3. Construction of a Human Corneal Endothelial Cell Sheet

### 3.1. Reconstruction of a Penetrating Keratoplasty Graft Using Cultured Human Corneal Endothelial Cells

During the past few decades, several laboratories have reported carriers or tissue-engineering composites for use in the construction of HCEC sheets. In the initial studies of cultured CECs, full-thickness corneal transplantations were performed in animal models with reconstructed corneal grafts bearing cultured animal CECs seeded on cat [56], rabbit [57,58,59], bovine [58,60], or murine [61] Descemet’s membranes or gelatin membranes [62,63]. Similar transplantation was performed in primates with cultured human neonatal and infant CECs [17,18,19].

The first attempt to construct CEC sheets using cultured CECs was performed by Gospodarowicz and Greenburg [56,57,58]. They seeded cultured bovine corneal endothelial cells onto bovine and rabbit corneas denuded of their endothelium. Subsequently, Engelmann’s laboratory developed a technique to seed cultured HCECs onto human corneas denuded of their own endothelium [20,22,23]. In 2001, Joyce’s laboratory reported a modified transplantation technique from an original method described by Gospodarowicz and Greenburg [24,56,57,58]. They seeded primary- or Passage 1-cultured HCECs onto the denuded Descemet’s membrane of a donor cornea. The recipient cornea was incubated in organ culture for as long as two weeks. The mean endothelial cell density in the transplanted corneas was 1895 cells/mm^2^ (range, 1503–2159 cells/mm^2^) [24].

Ishino and associates used amniotic membrane as a carrier for cultured HCEC transplantation [29]. They reported the density of the HCECs on the amniotic membrane was greater than 3000 cells/mm^2^. They transplanted the cultured HCEC sheets onto the rabbit cornea using PKP as follows. Circular HCEC sheets were placed on the endothelial side of an excised rabbit’s cornea after the stripping of Descemet’s membranes and these corneal buttons with circular HCEC sheets were transplanted by PKP. They reported that the transplanted cornea maintained corneal transparency for seven days after PKP using cultured HCEC sheets. We also succeeded in maintaining corneal transparency for six months in a rabbit model [25,26] and one month in a nude rat model [27] after PKP using the reconstructed full-thickness cornea with cultured HECEs.

### 3.2. Construction of a Human Corneal Endothelial Cell Sheet for a Descemet Stripping Automated Endothelial Keratoplasty Graft

The concept of these previous studies was based on the reconstruction of a full-thickness cornea using cultured HCECs because a full-thickness corneal transplantation technique had been mainly performed for CEC diseases such as Fuchs dystrophy and peudophakic or aphakic bullous keratopathy. However, this technique has frequent complications of high or irregular astigmatism, refractive error, and suture-related problems. Recently, DSAEK has become a standard procedure for corneal transplantation in patients with endothelial dysfunction [13,14,15,16]. This procedure improves postoperative visual function and reduces the risks associated with PKP, such as severe astigmatism and expulsive hemorrhage. In 2004, we had already established a method of transplanting only the cultured HCEC sheets before DSAEK was developed [28].

Here we introduce this new approach for treating CEC dysfunction using a cultured HCEC sheet for DSAEK. As the cell carrier, collagen sheets obtained from Nippi Research Institute of Biomatrix (Tokyo, Japan) were employed. These sheets were composed of a network of loosely cross-linked type I collagen fibers that had been treated with an alkaline solution, dried, and sterilized for 2 h under ultraviolet light [64,65]. Before use, the desiccated sheets were immersed in sterile saline for 10 min. A 6.0-mm trephine was used as the biopsy punch. Then 1.0 × 10^6^ HCECs in 300 µL of culture medium were transferred to sheets in each well of 96-well plates. The plates were centrifuged at 1000 rpm (176 × *g*) for 10 min to promote the attachment of cells to the sheets. After culturing for 2 days, nonadherent cells and debris were removed. Approximately 4000 100-mm culture dishes of confluent passage-5 HCECs can be produced from one donor cornea. And more than 10 HCEC sheets can be produced from one 100-mm dish. Therefore, we estimate that more than 1.0 × 10^3^ HCEC sheets can be made using the passage-5 HCECs from one donor cornea. Additionally, similar morphology in sheen in 5th passage HCECs was observed in HCECs after being maintained in culture dishes for at least 7 passages. Therefore we speculate that this method still result in the same success of engineering HCEC using at least 7th passage HCECs.

After our initial report, we developed an HCEC sheet using a thin human corneal stromal disc as a carrier of cultured HCEC sheets in 2009 [30]. Additionally, we reconstructed the HCEC sheets by seeding the cultured HCECs on various carriers such as decellularized thin-layer pig corneal stroma and human amniotic membrane (data not shown). These newly developed HCEC sheets showed similar morphology to the collagen-based HCEC sheets.

Nishida’s group reported a monolayer HCEC sheet that was cultured using novel temperature-responsive culture dishes onto which an ultrathin layer of temperature-responsive polymer, poly(N-isopropylacrylamide) (PIPAAm), is covalently grafted [66]. Nishida *et al.* commented that PIPAAm-grafted culture surfaces are slightly hydrophobic to facilitate cell adhesion under typical culture conditions at 37 °C because PIPAAm undergo a hydration phase transition across its lower critical solution temperature near 32 °C in water [66]. Because of the spontaneous and rapid hydration of the grafted PIPAAm, adherent cultured cells release spontaneously from these surfaces upon reducing culture temperature below the LCST without the need for proteolytic enzymes [66]. They had initially cultured HCECs on type IV collagen-coated dishes and seeded cultured HCECs onto the temperature-responsive culture dishes after several passages. The cells in their HCEC sheets showed primarily a hexagonal shape with numerous microvilli and cilia similar to the native corneal endothelium, by scanning electron microscopy. Lai and associates also cultured HCECs on a thermoresponsive type of PNIPAAm and seeded cultured HCECs onto gelatin hydrogel discs as a carrier of cultured HCEC sheets [32]. Choi and associates used decellularized thin-layer human corneal stroma as a carrier [31]. Liang and associates also developed a chitosan-based membrane made of hydroxyethyl chitosan, gelatin, and chondroitin sulfate as a new carrier of cultured HCEC sheets [34]. Additionally, Nishida and Tabata’s group recently developed gelatin hydrogels as carrier sheets for the reconstruction of HCEC sheets [33]. These newly proposed materials are very attractive candidate materials for use in future HCEC tissue regeneration.

### 3.3. Density of Cultured Human Corneal Endothelial Cells on Collagen Sheets

We succeeded in achieving a mean endothelial cell density >3000 cells/mm^2^ for HCECs cultured on collagen sheets by improving the cell seeding technique. Adhesion of the cells was promoted by centrifugation after seeding using the methods of Jamblatt [59] and Engelmann *et al*. [22,23] with modifications. Engelmann and associates recommended that each cell suspension be centrifuged at 33 × g for 5 min [22]. In our study, an HCEC suspension of 1.0 × 10^6^ cells in 300 µL culture medium was transferred to each sheet, and the sheet placed in individual wells of 96-well plates. The plates then were centrifuged at 1000 rpm (176 × g) for 10 min to enhance cell attachment to the sheets. Preoperative endothelial densities were around 1200 cells/mm^2^ without centrifugation, but the density was increased to about 3500 cells/mm^2^ by centrifuging the reconstructed corneal endothelial sheet. Application of fibronectin before cell seeding and a longer centrifugation time were found to prevent the detachment of HCECs from Descemet’s membrane.

### 3.4. Transport Activity of Human Corneal Endothelial Cell Sheets

**Figure 3:**
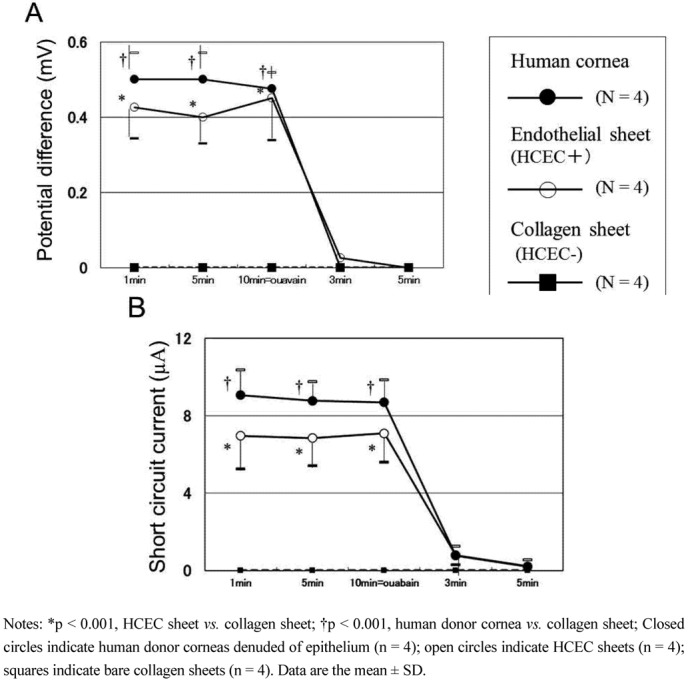
The pump functions in reconstructed HCEC sheets (arranged from reference [28] with permission). Potential difference (**A**) and short circuit current; (**B**) of human donor corneas (n = 4), cultured HCEC collagen sheets (n = 4), and bare collagen sheets (n = 4) are measured by an Ussing chamber. (**A**) The average potential difference for HCEC sheets is 80%–95% of that for human donor corneas denuded of epithelium. Addition of ouabain (an Na^+^-K^+^ ATPase inhibitor) causes the potential difference to become 0 mV in all samples tested; (**B**) The average short circuit current for HCEC sheets is 76%–82% of that for human donor corneas denuded of epithelium. Addition of ouabain causes the short circuit current to become 0 µA in all samples tested. The data were analyzed using one-way analysis of variance and the Scheffe’s multiple comparison test.

Corneal hydration and the consequential transparency depend primarily on sodium and bicarbonate ion transport, driven by the Na^+^/K^+^-adenosine triphosphatase (Na^+^/K^+^-ATPase) pump [67,68,69,70]. Therefore, the reconstructed HCEC sheets require transport activity to maintain corneal transparency after transplantation. We have examined the pump function in reconstructed HCEC sheets by electrophysiological measurements. The pump function of HCEC collagen sheets was measured in an Ussing chamber by the method reported previously with some modifications [71,72,73]. The donor corneas (n = 4), collagen sheets only (n = 4), and HCEC collagen sheets (n = 4) were mounted in the Ussing chamber. Corneas were incubated in Ringer solution containing 117.5 mM NaCl, 24 mM NaHCO_2_, 4 mM KCl, 1 mM Na_2_HPO_4_, 1 mM MgSO_4_, 4.45 mM glucose, 1 mM reduced glutathione and 2.54 mM CaCl_2_, and bubbled with a 5% CO_2_–7% O_2_, 88% N_2_ gas mixture to pH 7.38. After steady state levels of the potential difference and short circuit current were reached, 0.1 mM ouabain, an Na^+^,K^+^-ATPase inhibitor, was added to the chamber and the potential difference and short circuit current were redetermined. The mean potential difference of the HCEC collagen sheets was 85% at 1 min, 80% at 5 min and 95% at 10 min of that for human donor corneas (Figure 3A). The average short circuit current of the HCEC collagen sheets was 76% at 1 min, 78% at 5 min and 82% at 10 min of that for human donor corneas denuded of epithelium. The potential difference and short circuit currents of the collagens sheets and human donor corneas denuded of epithelium and endothelium were 0 mV and 0 A at each time of assessment (Figure 3A and Figure 3B). After the Na^+^, K^+^-ATPase inhibitor ouabain was added to the chambers, the potential difference and short circuit currents reached 0 mV for all test samples within 5 min. These results indicate that CEC pump function, which mainly depends on Na^+^, K^+^-ATPase, was satisfactory with our HCEC sheets.

## 4. Transplantation of Descemet Stripping Automated Endothelial Keratoplasty Grafts Using Cultured Human Corneal Endothelial Cells

### 4.1. Transplantation of Human Corneal Endothelial Cell Sheets Using Collagen as a Carrier in a Rabbit Model

We described the first application of cultured HCEC sheets for DSAEK grafts in 2004 [28]. In the study, we used a collagen sheet as a carrier for cultured HCEC transplantation. In our second study, DSAEK grafts were produced by seeding cultured HCEC suspensions onto thin-layer human corneal stromal discs [30]. These DSAEK grafts were transplanted into rabbit corneas and HCECs seeded on DSAEK grafts succeeded in maintaining a morphology similar to HCECs *in vivo* and contributed to reduce corneal edema in an animal model.

### 4.2. Other Attempts to Transplant Cultured Corneal Endothelial Cell Sheets in Animal Models

Several other research groups also reported developing DSAEK grafts using cultured CECs (Table 1). Lai and associated transplanted HCEC sheets cultured on PNIPAAm and gelatin-based material into rabbit eyes and found that corneal transparency was restored within two postoperative weeks [32]. Koizumi and associates reconstructed monkey CEC sheets by the cultivation of monkey CECs on collagen type I carriers for four weeks [74,75]. They transplanted the reconstructed monkey CEC sheets into monkey eyes using the DSAEK technique. The transplanted sheets were detached from the host corneas one week after grafting, but the corneas recovered their clarity in the surgical eyes that received cultivated monkey CEC sheet transplants six months after transplantation.

**Table 1:** Summary of transplantation technique of cultured corneal endothelial cell sheets in animal models.

Author	Species of cultured CEC	Cell carrier	Host animal	Transplantation technique	Journal (Year)
Mimura	Human	Collagen	Rabbit	DSAEK	*Invest Ophthalmol* *Vis Sci* (2004)
Ishino	Human	Amniotic membrane	Rabbit	PKP	*Invest Ophthalmol* *Vis Sci* (2004)
Sumide	Human	PNIPAAm	Rabbit	PKP	*FASEB J* (2006)
Lai	Human	PNIPAAm and gelatin	Rabbit	DSAEK	*Transplantation* (2007)
Koizumi	Monkey	Collagen	Monkey	DSAEK	*Invest Ophthalmol Vis Sci* (2007)
Honda	Human	Thin human corneal stromal disc	Rabbit	DSAEK	*Arch Ophthalmol* (2009)

Notes: CEC, Corneal Endothelial Cell; PKP, Penetrating Keratoplasty; DSAEK, Descemet Stripping Automated Endothelial Keratoplasty.

### 4.3. Transplantation Technique of Human Corneal Endothelial Cell Sheets with Descemet Stripping Automated Endothelial Keratoplasty

**Figure 4:**
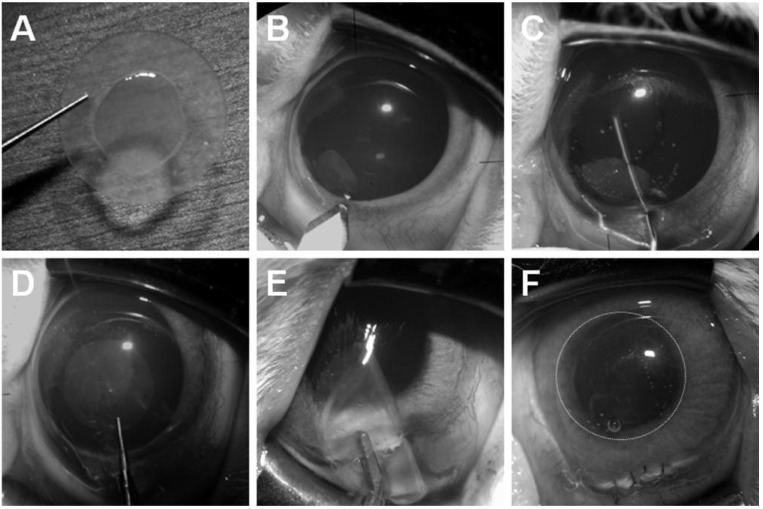
Procedure for HCEC sheet transplantation. (**A**) HCEC collagen sheet on a silicon plate; (**B**) A 6-mm sclerocorneal incision centered at 12 o’clock is made with a slit-knife;(**C**) A 6.0-mm diameter circular Descemetorhexis was performed with a 30-gauge needle and Descemet’s membrane was removed from the anterior chamber; (**D**,**E**,**F**): (**D**) The HCEC sheet was inserted into the anterior chamber using the forceps; or (**E**)a foldable silicone plate; and (**F**) attached to the posterior stroma.

Here, we shall introduce our experimental DSAEK model using cultured HCEC sheets (Figure 4) and then describe empirical observations after the transplantation of a DSAEK graft. In our first transplantation of a DSAEK graft using culture HCECs, a 6-mm sclerocorneal incision centered at 12 o’clock was made with a slit knife, a circular descemetorhexis (6.0-mm in diameter) was created in the center of the rabbit cornea with a 30-gauge needle, and Descemet’s membrane was removed from the anterior chamber. A circular HCEC sheet (with the HCEC side up) was brought into the anterior chamber and then was fixed to the posterior stroma that had been stripped of Descemet’s membrane. If the sheet was difficult to attach, an air bubble was injected into the anterior chamber. The sclerocorneal wound was closed with two or three interrupted sutures of 10–0 nylon. This transplantation technique is almost the same as that in the clinically performed DSAEK procedure. The rabbits were divided into either the DSAEK group (rabbits with peeling of Descemet’s membrane and transplantation of an HCEC sheet) or the control group (rabbits with peeling of Descemet’s membrane only). Each group comprised of four rabbits (four eyes). No immunosuppressive agents were administered either topically or systemically.

### 4.4. Observation after Transplantation

**Figure 5:**
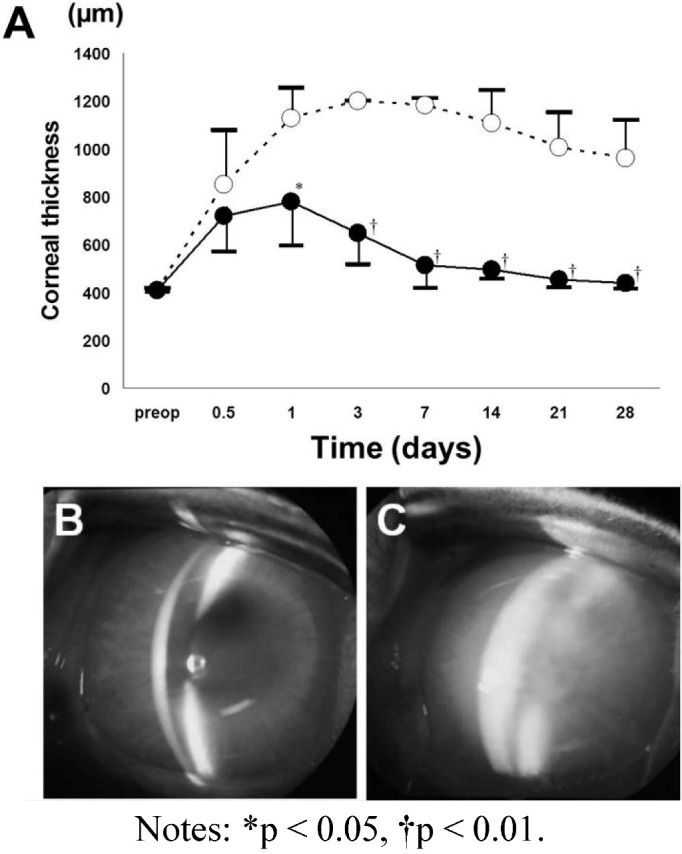
Central corneal thickness (**A**) and anterior segment photographs (**B**,**C**) after transplantation of a DSAEK graft reconstructed with collagen and cultured HCECs [28]. The DSAEK graft using cultured HCECs and collagen (DSAEK group) or a bare collagen sheet (control group) are transplanted into rabbit corneas after stripping of the Descemet membrane. In the control group (open circles), the mean corneal thickness remains at around 1000 m for 28 days. In contrast, the mean corneal thickness gradually decreases in the DSAEK group (closed circles) and becomes significantly less than in the control group. There are significant differences of corneal thickness between the DSAEK and control groups on Days 1, 3, 7, 14, 21 and 28 using an unpaired t-test. (**B**) Representative anterior segment photographs obtained with a slit-lamp microscope at 28 days after surgery show a thin cornea without stromal edema in the DSAEK group; (**C**) while severe corneal edema is observed in the control group.

Corneal edema decreased much earlier after HCEC sheet transplantation in the DSAEK group than in the control group. In the control group, mean corneal thickness remained at approximately 1000 µm throughout the 28-day observation period. In contrast, corneal edema decreased rapidly in the DSAEK group and the cornea was significantly thinner than in the control group at 1 (*P* < 0.05, unpaired t-test), 3, 7, 14, 21 and 28 days (*P* < 0.001, unpaired t-test) after surgery. Figure 5 shows representative anterior segment photographs from each group. The cornea is opaque with severe stromal edema in the control group, while the cornea transplanted with a cultured HCEC sheet is clear and has no stromal edema on Day 28 in the DSAEK group (Figure 5). In the DSAEK group, grafts remained transparent for one month after surgery and the corneas with HCEC sheets were significantly thinner than the corneas of the control group. These results suggest the feasibility of performing corneal reconstruction by using HCECs cultured from adult donor corneas.

### 4.5. Histologic Examination

Fluorescence microscopy of whole mounted corneas showed DiI-positive cells localized on the transplanted collagen sheet and a clear margin of the sheet at 28 days after transplantation. The HCECs on the collagen sheets had a fairly regular morphology with well-defined boundaries. Most cells on the collagen sheets transplanted to the posterior surface of the cornea were DiI-positive in the DSAEK group analyses; details can be found in the original article [28]. Because endocytosed DiI cannot be transferred to adjacent cells [76], it is probable that the cultured HCECs remained on the sheet. The endothelial cell density of the four grafts in the DSAEK group was around 2500 cells/mm^2^ at 28 days after surgery, whereas the preoperative endothelial density was around 3500 cells/mm^2^. In the control group, no CECs were detected on the stroma at the site of the descemetorhexis.

**Figure 6:**
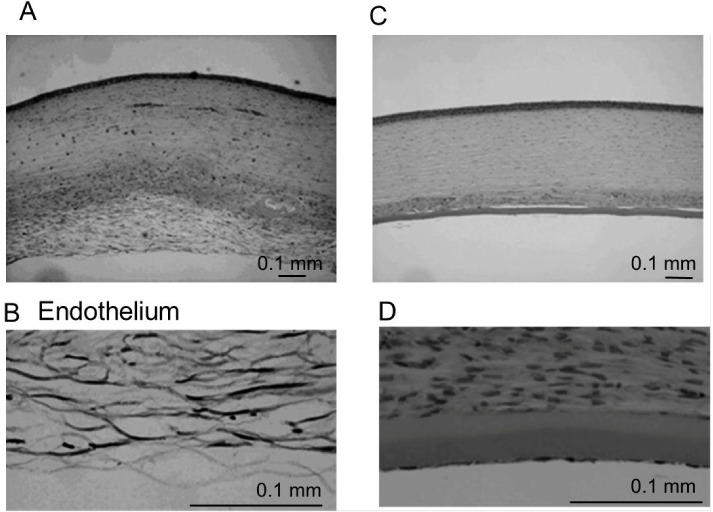
Histological examination of the cornea at 28 days after surgery. (**A**,**B**) In the control group, HCECs and Descemet’s membrane are absent. Severe stromal edema and diffuse cell infiltration are observed; (**C**,**D**) Hematoxylin-eosin staining shows a collagen sheet with HCECs on the posterior surface of the cornea and no stromal edema in the DSAEK group. Fibroblast-like cells are detected in the posterior corneal stroma attached to the collagen sheet.

Hematoxylin-eosin-stained sections obtained 28 days after transplantation are shown in Figure 6. There is edema and diffuse cellular infiltration of the stroma in the control group (Figure 6A). Fibrous tissue and fibroblast-like cells were observed in the posterior stroma of the control group (Figure 6B). In contrast, there was no edema of the transplanted HCEC collagen sheets in the DSAEK group (Figure 6C,D ).

The study of the transplantation of DSAEK graft using the thin-layer human corneal stromal discs as a carrier for cultured HCEC transplantation showed almost the same results that were seen in this study [30]. Furthermore, we transplanted the bioengineered DSAEK graft using cultured HCECs and human amniotic membrane into rabbits’ cornea and this DSAEK model using an amniotic membrane as a carrier also showed rapid recovery of corneal edema without possible side effects such as intraocular pressure increase and graft rejection (data not shown). In these various DSAEK models using cultured HCECs, HCEC grafts remained transparent for one month after surgery and the HCEC sheets were significantly thinner than the corneas of the untransplanted control group. These results suggest the feasibility of performing corneal reconstruction by using HCECs cultured from adult donor corneas.

## 5. Conclusion

New techniques that can replace full-thickness corneal transplantation have been tried both clinically and experimentally. DSAEK surgery has rapidly replaced conventional PKP as the preferred procedure for the treatment of endothelial disorders. This paper has highlighted some of the latest experimental innovations in DSAEK surgery using cultured HCEC sheets to overcome donor organ shortage. The novel techniques presented here are good candidates for future treatments of CEC dysfunction. Autologous CEC transplantation is undoubtedly an ideal strategy to completely negate the possibility of rejection. Because CECs from the peripheral cornea contain a higher density of precursors than CECs from the central cornea in rabbits [77] and humans [78], cultures of peripheral cells obtained by resecting a small piece of Descemet’s membrane may eventually allow HCEC sheet transplantation for unilateral bullous keratopathy. Cultured HCECs should become a powerful tool for cell or tissue repair and regeneration of corneal endothelium.
